# Anticoagulant rodenticide intoxication in east China: a three-year analysis

**DOI:** 10.1080/20961790.2016.1242042

**Published:** 2016-12-14

**Authors:** Hui Yan, Lin Zhu, Xianyi Zhuo, Min Shen, Ping Xiang

**Affiliations:** Department of Forensic Toxicology, Shanghai Key Laboratory of Forensic Medicine, Shanghai Forensic Service Platform, Institute of Forensic Science, Ministry of Justice, PRC, Shanghai, China

**Keywords:** Forensic science, forensic toxicology, anticoagulant rodenticides, intoxication, bromadiolone, brodifacoum, poisoning

## Abstract

The study was designed to analyze the incidence and pattern of anticoagulant rodenticide intoxication in east China and to discuss strategies of diagnosis based on laboratory analysis experience. A total of 117 patients with anticoagulant rodenticide poisoning confirmed by toxicological analysis in east China were included in this study from 2011 to 2013. The mean concentration of anticoagulant rodenticide, age, and gender of these patients, as well as the mode and type of poisoning, were discussed. The age ranged from less than 1 to 80 years with the feeble preponderance of males (M = 53.0%, F = 47.0%). The 0–9 age group covered the largest ratio of these anticoagulant rodenticide poisoning patients. Accidental or voluntary ingestion seems to be the most common cause of intoxication, with still the poisoning cause being unknown for a large number of positive analyses. Bromadiolone was the most commonly observed anticoagulant rodenticide found in the biological samples, followed by brodifacoum. The concentrations of bromadiolone and brodifacoum that were detected in the first collected whole blood from each patient ranged from 1 to 878 ng/mL (mean 97.9 ng/mL) and from 0.5 to 1566 ng/mL (mean 225.1 ng/mL), respectively. The data analysis shows a high incidence of anticoagulant rodenticide poisoning without awareness of the poisoned subjects, clearly emphasizing the need for toxicological analysis in patients with vitamin K-dependent coagulation disorder and restriction on availability of anticoagulant rodenticide.

## Introduction

Anticoagulant rodenticides, which can be divided into first-generation and second-generation compounds, are widely used in agricultural and urban rodent control. The second-generation anticoagulant rodenticides (SGARs) with greater toxicity and persistent to animals and human beings are active after single dose [1–3]. In humans, the half-life varies between 243 and 1656 hours for brodifacoum and between 17 and 37 hours for warfarin [4]. The United States Environmental Protection Agency banned most residential uses of SGARs from 2011 [5]. Their use remains legal for agricultural, industrial and some commercial applications in the United States and are widely used in many other countries.

Anticoagulant rodenticides act as vitamin K antagonists, frequently cause poisoning by accidental exposure of sublethal amounts of rodenticides, which leads to abnormal coagulopathy or bleeding and results in severe clinical outcomes [6]. Bromadiolone and brodifacoum are the most commonly used SGARs in China, and thus involved in the majority of anticoagulant rodenticide poisoning cases. From 2005 to 2010, 46 anticoagulant rodenticide poisoning patients were treated in a Henan Province hospital [7]. From July 2008 to April 2011, 12 patients were diagnosed with anticoagulant rodenticide occult poisoning in a Changsha hospital [8]. In 2009, 176 middle-school students in Leqing were sent to hospital after consuming food that had been accidently contaminated with bromadiolone [9].

The diagnosis and treatment of anticoagulant rodenticides poisoning is still challenging in spite of special vitamin K therapy, when there is no significant history of exposure or ingestion [10,11]. The poisoning patterns differ regionally as a result of local variations in environmental, cultural, and religious situations. Knowing the specific pattern of poisoning in a region is important for aiding local governments in establishing effective poisoning prevention and treatment programmes.

Low-level bodily residues require high sensitivity in the detection method used for SGARs, and the forensic or clinical laboratories benefit a lot from the appearance of liquid chromatography-tandem mass spectrometry (LC–MS/MS) [12]. Vindenes et al. [13] developed a method for quantification of bromadiolone in whole blood, using liquid chromatography–mass spectrometry (LC–MS). The limit of detection (LOD) was 5 ng/mL, and the limit of quantification was 10 ng/mL. Schaf and Montgomery [14] identified bromadiolone, brodifacoum, coumachlor, coumatetralyl, difenacoum and warfarin in whole blood specimens with a LOD of 10 ng/mL. Fourel et al. [15] developed a new LC–MS/MS ion-trap technique for the simultaneous determination of 13 anticoagulant rodenticides in plasma with LODs that ranged from 5 to 25 ng/mL.

The primary purpose of this study was to analyze the incidence and pattern of anticoagulant rodenticide (bromadiolone, brodifacoum, coumatetralyl, chlorohacinone, diphacinone, valone and pindone, coumafuryl, difenacoum, flocoumafen, warfarin, coumachlor and dicumarol) intoxication in east China and to discuss strategies of diagnosis based on laboratory analysis experience.

## Methods

### Chemicals and reagents

Bromadiolone, brodifacoum, coumatetralyl, chlorohacinone, diphacinone, valone and pindone were purchased from AccuStandard (New Heaven, USA). Coumafuryl, difenacoum, flocoumafen, warfarin and warfarin-d5 (IS, 100 ng/mL in acetonitrile) were purchased from Dr Ehrenstorfer GmbH (Augsburg, Germany). Coumachlor and dicumarol were purchased from Toronto Research Chemicals Inc. (Ontario, Canada). Methanol (HPLC grade) was purchased from Sigma-Aldrich (St. Louis, MO, USA), and ammonium acetate (HPLC grade) was purchased from Fluka (Buchs, Switzerland). Deionized water was obtained from a Milli-Q water purification system (Millipore, MA, USA). All other organic reagents were of analytical-reagent grade.

### Samples

Information on 117 anticoagulant rodenticide poisoning patients in east China were collected from the Department of Forensic Toxicology, Institute of Forensic Science, Ministry of Justice, PRC (IFS) between the years 2011 and 2013. The first collected blood of 108 patients and washed gastric juice of the other nine patients were tested positive for anticoagulant rodenticide.

### UPLC–MS/MS conditions

The concentrations of the anticoagulant rodenticides in the whole blood were determined by previously published LC–MS/MS methods [16]. A 4000 Q TRAP mass spectrometer equipped with an ESI source (Applied Biosystems/MDS SCIEX, Toronto, Canada) and an Acquity Ultra Performance Liquid Chromatography (Waters, Milford, MA, USA) instrument were used for the LC–MS/MS analysis. The mass instrument was operated in the negative electrospray ionization mode with a multiple reaction-monitoring (MRM) using an ion spray voltage of −3.5 kV and a source temperature of 500 °C. Nitrogen was used as a nebulizing gas (GS 1, 60 psi), turbo spray gas (GS 2, 65 psi) and curtain gas (25 psi). The collision-activated dissociation (CAD) was set to a medium level. Analyst 1.5 software was used for the instrument control and data acquisition. The separation was performed on a XBridge C18 column (5 μm particle size, 50 mm × 2.1 mm i.d., Waters, Ireland) using an LC mobile phase gradient at a constant flow rate of 200 μL/min.

### Sample preparation

A total of 10 μL of IS working solution and 3 mL of ethyl acetate were added to 1 mL of whole blood or urine. After vortex mixing for 1 min, the mixtures were centrifuged at 2500 rpm for 3 min. The organic layer was transferred to 5-mL glass tubes and evaporated to dryness at 55 °C. The residue was reconstituted in 100 μL of methanol, and 5 μL of the residue was injected into the LC–MS/MS system.

## Results

### Number of cases

During the study period (2011–2013), there were 177 anticoagulant rodenticide analysis requests made to IFS. In 2011, in a total of 39 analysis requests, 29 cases (74.4%) were positive. In 2012, the analysis requests increased to 56 as well as the positive cases (35,62.5%), and in 2013, the total cases analyzed also increased to 82, with 53 positive cases (64.6%) (Table 1). The cause of intoxication was provided in the toxicological analysis request form. 31.6% of the 117 positive analyses had indication of intoxication suspicion, 19.7% of suicide, 0.8% of homicide, and 12.0% of accident. However, 62.5% of the positive analyses have no information at all about the possible cause of intoxication. Most of the anticoagulant rodenticide analyses were requested by hospitals and just one case was sent by police.

**Table 1.** Anticoagulant rodenticide intoxication cases confirmed by LC-MS analysis between 2011 and 2013.

		Causes [*n* (%)]				Positive cases [*n* (%)]	
Year	Total (*n*)	Suicide	Homicide	Accident	Unknown	Male	Female
2011	29	8 (27.6)	0 (0.0)	5 (17.2)	16 (55.2)	15 (51.7)	14 (48.3)
2012	35	7 (20.0)	1 (2.9)	3 (8.6)	24 (68.6)	21 (60.0)	14 (40.0)
2013	53	8 (15.1)	0 (0.0)	6 (11.3)	39 (73.6)	26 (49.1)	27 (50.9)
Total	117	23 (19.7)	1 (0.8)	14 (12.0)	79 (67.5)	62 (53.0)	55 (47.0)

### Gender and age distribution

**Figure 1.**
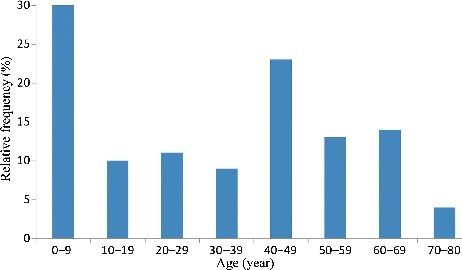
Age distribution of anticoagulant rodenticide poisoning (*N*=117).

During the three years analysis, there were 62 male patients and 55 female ones. The ratio was 1.1. The mean age in the anticoagulant rodenticide poisoning cases was 32.2 years (range from 0.7 to 80 years). According to Figure 1, peak incidence was observed in the age group of 0–9 years (33 cases, 28.2%), and the lowest incidence was observed in the age group 70–80 years (4 cases, 3.4%). The children are easily been attracted by the bright colour of anticoagulant rodenticide, for example, the warning colours of bromadiolone and brodifacoum were red and blue, respectively. It was found that patients in the age group of 40–49 years account for the second highest proportion of poisoning cases (19.7% of 117 positive cases). It may indicate population between the ages 40 and 49 years was the main labour engaged in agriculture activities.

### Detected rodenticides

**Figure 2.**
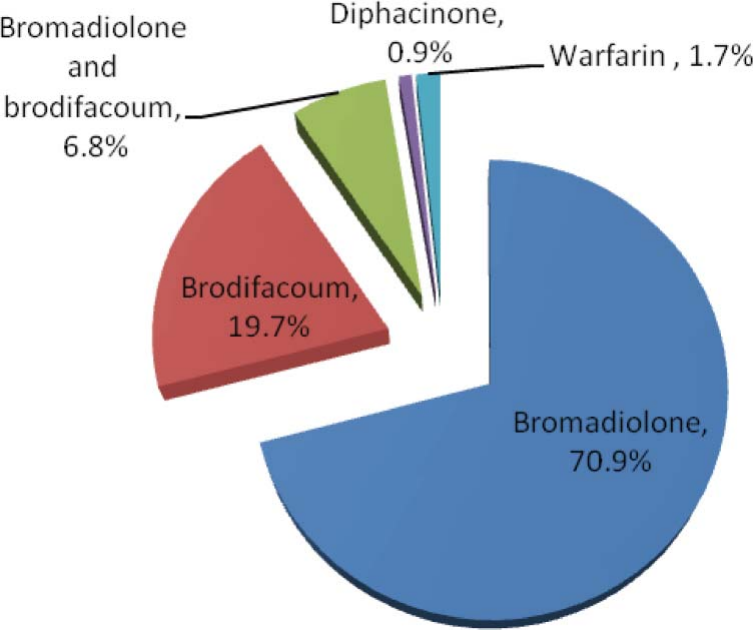
Incidence of pesticide poisoning.

Poisoning cases due to anticoagulant rodenticide (*n* = 117) were reviewed retrospectively (Figure 2). A total of four kinds of anticoagulant rodenticide were shown altogether. The most frequently encountered anticoagulant was bromadiolone (70.9%), followed by brodifacoum (19.7%). Both bromadiolone and brodifacoum were detected in 6.8% of the positive anticoagulant rodenticide analyses. It was found that bromadiolone and brodifacoum accounted for the majority of the positive cases (97.4%). The mean concentrations of bromadiolone and brodifacoum that were detected in the first collected whole blood were 97.9 and 225.1 ng/mL, respectively. Warfarin was found in blood samples of two patients with (0.4 and 2.4 μg/mL) concentration not exceeding therapeutic concentration (1.0–3.1 μg/mL) [17]. The results of urine test were consistent with those of blood analysis. Urine samples of 34 patients with bromadiolone poisoning were collected and tested for anticoagulant rodenticides. Bromadiolone was not detected in urine samples while blood samples of all the 34 patients showed positive analyses. Similarly, no brodifacoum was detected in urine samples of eight patients with positive analytical results of blood brodifacoum. It is probably because bromadiolone and brodifacoum were eliminated mostly via faeces. Blood samples from 10 patients were collected and monitored continuously with different sampling times until the blood bromadiolone concentration fell below 0.1 ng/mL (Table 2). Although the elimination half-lives of bromadiolone was long, it was still difficult to explain that it took two years and one month for a male patient to eliminate bromadiolone from blood (less than 0.1 ng/mL). Repeated exposure of bromadiolone was highly suspected although it was denied by the patient.

**Table 2.** Information of ten patients with bromadiolone poisoning.

No.	Age (year)	Gender	Peak concentration (ng/mL)	Elimination time (month)	Clinical symptoms
1	57	M	770	25	Gingival bleeding and systemic purple skin
2	28	M	18.1	12	Hematuria and flank pain
3	4	F	83	4	Systemic ecchymoses
4	2	F	0.5	1	Repetitive mucocutaneous bleeding and gingival bleeding
5	78	F	27	2	Hematuria and sudden syncope after melaena
6	43	F	5.3	2	Gingival bleeding
7	67	M	5.5	7	Repetitive ecchymoses and gingival bleeding
8	60	M	168	18	Repetitive gingival bleeding and hematuria
9	6	M	16	9	Systemic scattered petechiae, ecchymoses
10	2	M	37	8	Gingival bleeding

## Discussion

Anticoagulant rodenticides are now very commonly used rodenticides since the use of tetramethylene disulfotetramine has been forbidden in the 1970s in China [18]. Poisoning with anticoagulant rodenticides has increased over the past years and has become a growing public health problem [4,13,19]. Compared with the rapid onset of symptoms from tetramethylene disulfotetramine exposure, the SGARs have prolonged anticoagulant effect by inhibiting the carboxylation of vitamin K-dependent factors (II, VII, IX, and X) in the liver [13]. In health care facilities, the activated partial thromboplastin time (APTT), prothrombin time (PT), and international normalized ratio (INR) are used for anticoagulant intoxication screening [16]. These hemostatic parameters might be influenced by several diseases other than by anticoagulant ingestion, which results in a delayed suspicion of anticoagulant rodenticides.

Compared with adults, children (0–9 years old) account for a higher proportion of poisoning cases (28.2% of 117 positive cases). Rodent bait products are composed of common foods such as grain, corn, peanuts, and instant noodles, produced in bright colours with an active rodenticide poison additive. Many children do not know which substances are poisonous, and hand-to-mouth contact is typical in children. Although rodent bait is placed in corners of rooms, floor dust might be contaminated. In crawling babies, ingestion of rodenticide probably results from hand-to-mouth transfer because of inadequate hygienic conditions. It is a common social phenomenon that young children are frequent victims of unintentional exposure to poisons. Over 16000 cases of superwarfarin intoxication are reported annually by the Poison Control Center's toxic exposure surveillance system in the United States [20]. The majority of cases are related to accidental ingestion, predominantly in children under six years old. In a survey of acute poisoning in South Africa, there was a preponderance of accidental exposure in children (98.8%), and there were more cases in males (59.7%) than in females. The Lyon Poison Control Center reported that the majority of the cases of anticoagulant exposure in 2010 occurred in children, especially in those between 1 and 4 years of age [21].

Twenty-three suicide patients out of 117 positive analyses (19.7%) were between 21 and 73 years old, without age preference. The source of ingestion was unknown in 69 of the 117 patients. It is difficult to state the possible poisoning cause for children. Moreover, the majority of these patients had in common that they frequented concession stands without certification for food. More stringent legislation and enforcement regarding the sale and distribution of anticoagulant rodenticides are needed. Most of the patients went to hospital for hemorrhagic symptoms and were informed of intoxication via toxicological analysis report. Treatment of these patients is typically difficult because of the lack of a significant exposure or ingestion history and high possibility of repeated poison exposure. The high incidence of anticoagulant rodenticide poisoning without awareness of the poisoned subjects clearly emphasized the need for toxicological analysis in patients with vitamin K-dependent coagulation disorder. Very low mortality (one case of 117) was associated with anticoagulant rodenticide use. In fact, various formulations used for rodent control have low concentration (0.005% usually) of the active compound.

Bromadiolone and brodifacoum were the most frequently detected anticoagulant rodenticide, since they are widely used to control rodents in China. The concentrations of bromadiolone and brodifacoum detected in the first collected whole blood ranged from 1 to 878 ng/mL and from 0.5 to 1566 ng/mL, respectively. All of the patients except one survived and were treated with oral or intravenous vitamin K_1_. Higher blood brodifacoum levels were reported in the literature [22–25]. The highest brodifacoum concentrations were detected in bile (4276 ng/mL) and femoral blood (3919 ng/mL) in a fatal poisoning [22]. Olmos presented the case of a 46-year-old woman who survived after brodifacoum poisoning [23]. Five days after admission, the serum brodifacoum level was 1302 ng/mL. To our knowledge, a blood bromadiolone concentration of 878ng/mL is the highest reported level. Vindenes et al. reported that a 62-year-old woman poisoned with bromadiolone had a peak blood level of 750 ng/mL [13].

Sometimes it is difficult to identify the exposure time clearly because of the delayed onset of symptoms. Serial plasma levels might help to determine the approximate stage of poisoning [26]. A relatively rapid drop associated with a short half-life indicates a quite recent exposure, whereas a much slower decline with a longer half-life suggests a more distant contact. A surge at any time in daily or bi-daily measurement suggests repeated exposure, which the clotting profile alone cannot reveal, especially during vitamin K_1_ therapy. Explicit diagnosis of anticoagulant rodenticide poisoning could help to isolate from poison and identify the endpoint of therapy. After tracing blood bromadiolone in 10 patients, it was found that the peak concentration of bromadiolone often appeared in the first collected blood sample and the elimination time ranged from 1 to 25 months. Bromadiolone distribution in the body might follow at least a two-compartment model because of lipophilicity. The half-lives of bromadiolone in human blood varied from 50 h to 13 days [13,27,28]. The serum brodifacoum level was 210 ng/mL 5 weeks after ingestion [29]. The SGARs have longer half-lives than the first-generation anticoagulant rodenticides such as warfarin [4]. For SGARs intoxication, a regimen combining the coagulation profile and blood level monitoring is a good strategy for the endpoint of vitamin K_1_ therapy. Lo [26] proposed that a plasma bromadiolone level of 10 ng/mL could be used as one of the logical and safe therapeutic endpoints for vitamin K_1_ therapy in bromadiolone and brodifacoum intoxication. More than 24% of the bromadiolone and brodifacoum concentrations in the first collected whole blood were lower than 10 ng/mL. However, it was suggested by Kanabar and Volans that less treatment in children with accidental superwarfarin poisoning is better [30]. More data are necessary on the metabolism of SGARs (including inter-individual variability) before the blood level could serve as a logical and safe therapeutic endpoint for vitamin K_1_ therapy.

## Conclusion

The present study shows that anticoagulant rodenticide poisoning is a growing public health problem in China. Bromadiolone is the most frequently encountered SGAR, and children (0–9 years old) account for a higher proportion of poisoning cases. Testing for anticoagulant rodenticides is recommended in patients with a vitamin K_1_-dependent coagulation disorder, helping to diagnose poisoning in time and to identify the endpoint of vitamin K_1_ therapy. Further regulatory enforcement should be carried out by the government to restrict and manage the use of anticoagulant rodenticide to avoid unconscious anticoagulant rodenticide poisoning.

## Compliance with Ethical Standards

The authors declare that they have no conflict of interest. All procedures performed in studies involving human participants were in accordance with the relevant national legislation and local guidelines.

## References

[1] Gallocchio F, Basilicata L, Benetti C, et al. Multi-residue determination of eleven anticoagulant rodenticides by high-performance liquid chromatography with diode array/fluorimetric detection: investigation of suspected animal poisoning in the period 2012–2013 in north-eastern Italy. Forensic Sci Int. 2014;244:63–69. 25195128 10.1016/j.forsciint.2014.08.012

[2] Berny P, Velardo J, Pulce C, et al. Prevalence of anticoagulant rodenticide poisoning in humans and animals in France and substances involved. Clin Toxicol (Phila). 2010;48:935–941. 21171851 10.3109/15563650.2010.533678

[3] Vandenbroucke V, Bousquet-Melou A, De Backer P, et al. Pharmacokinetics of eight anticoagulant rodenticides in mice after single oral administration. J Vet Pharmacol Ther. 2008;31:437–445. 19000263 10.1111/j.1365-2885.2008.00979.x

[4] Spahr JE, Maul JS, Rodgers GM Superwarfarin poisoning: a report of two cases and review of the literature. Am J Hematol. 2007;82:656–660. 17022046 10.1002/ajh.20784

[5] Peterson ME Bromethalin. Top Compan Anim Med. 2013;28:21–23. 10.1053/j.tcam.2013.03.005 23796484

[6] De Paula EV, Montalvao SAL, Madureira PR, et al. Simultaneous bleeding and thrombosis in superwarfarin poisoning. Thromb Res. 2009;123:637–639. 18372007 10.1016/j.thromres.2008.02.004

[7] Wang Z, Liu Z, Zhang Y, et al. Acquired deficiency diseases with vitamin k-dependent coagulation factor caused by anticoagulant rodenticides poisoning: clinical analysis of 46 cases. Chin J Thromb Hemost (Chinese). 2011;17:166.

[8] Cao X, Li L, Zheng Y Clinical analysis of 12 patients caused by long-acting anticoagulant rodenticide occult poisoning. Zhong Nan Da Xue Xue Bao Yi Xue Bao (Chinese). 2012;37:849. 10.3969/j.issn.1672-7347.2012.08.016 22954910

[9] Wang X, Chen X Diagnosis and treatment of 176 patients with bromadiolone poisoning. Chin Gen Pract (Chinese). 2010;13:1674.

[10] Jin BC, Guang YG, Wang TC, et al. Anticoagulant rodenticide poisoning: 21 misdiagnosis case. Guizhou Med J (Chinese). 2012;36:641–642.

[11] Chua JD, Friedenberg WR Superwarfarin Poisoning. Arch Intern Med. 1998;158:1929–1932. 9759690 10.1001/archinte.158.17.1929

[12] Peters FT Recent advances of liquid chromatography-(tandem) mass spectrometry in clinical and forensic toxicology. Clin Biochem 2011;44:54–65. 20709050 10.1016/j.clinbiochem.2010.08.008

[13] Vindenes V, Karinen R, Hasvold I, et al. Bromadiolone poisoning: LC-MS method and pharmacokinetic data. J Forensic Sci. 2008;53:993–996. 18482378 10.1111/j.1556-4029.2008.00737.x

[14] Schaff JE, Montgomery MA An HPLC-HR-MS-MS method for identification of anticoagulant rodenticides in blood. J Anal Toxicol. 2013;37:321–325. 23667199 10.1093/jat/bkt036

[15] Fourel I, Hugnet C, Goy-Thollot I, et al. Validation of a new liquid chromatography- tandem mass spectrometry ion-trap technique for the simultaneous determination of thirteen anticoagulant rodenticides, drugs, or natural products. J Anal Toxicol. 2010;34:95–102. 20223102 10.1093/jat/34.2.95

[16] Yan H, Xiang P, Zhu L, et al. Determination of bromadiolone and brodifacoum in human blood using LC-ESI/MS/MS and its application in four superwarfarin poisoning cases. Forensic Sci Int. 2012;222:313–317. 22910058 10.1016/j.forsciint.2012.07.008

[17] Winek CL, Wahba WW, Winek CL Jr, et al. Drug and chemical blood-level data. Forensic Sci Int. 2001;122:107–123. 11672964 10.1016/s0379-0738(01)00483-2

[18] Wu Y, Sun C Poison control services in China. Toxicology. 2004;198:279–284. 15138053 10.1016/j.tox.2004.02.003

[19] Rauch AE, Weininger R, Pasquale D, et al. Superwarfarin poisoning: a significant public health problem. J Commun Health. 1994;19:55–65. 10.1007/BF02260521 8169251

[20] Altay S, Cakmak HA, Boz GC, et al. Prolonged coagulopathy related to coumarin rodenticide in a young patient: superwarfarin poisoning. Cardiovasc J Afr. 2012;23:e9–e11. 10.5830/CVJA-2012-051 23108575

[21] Berny P, Velardo J, Pulce C, et al. Prevalence of anticoagulant rodenticide poisoning in humans and animals in France and substances involved. Clin Toxicol. 2010;48:935–941. 10.3109/15563650.2010.533678 21171851

[22] Palmer RB, Alakija P, de Baca JE, et al. Fatal brodifacoum rodenticide poisoning: autopsy and toxicologic findings. J Forensic Sci. 1999;44:851–855. 10432620

[23] Olmos V, López CM Brodifacoum poisoning with toxicokinetic data. Clin Toxicol (Phila). 2007;45:487–489. 17503253 10.1080/15563650701354093

[24] Hollinger BR, Pastoor TP Case management and plasma half-life in a case of brodifacoum poisoning. Arch Intern Med. 1993;153:1925–1928. 8250654

[25] Gunja N, Coggins A, Bidny S Management of intentional superwarfarin poisoning with long-term vitamin K and brodifacoum levels. Clin Toxicol (Phila). 2011;49:385–390. 21740137 10.3109/15563650.2011.587126

[26] Lo VM, Ching CK, Chan AY, et al. Bromadiolone toxicokinetics: diagnosis and treatment implications. Clin Toxicol (Phila). 2008;46:703–710. 19238731 10.1080/15563650701504366

[27] Jin MC, Ren YP, Xu XM, et al. Determination of bromadiolone in whole blood by high-performance liquid chromatography coupled with electrospray ionization tandem mass spectrometry. Forensic Sci Int. 2007;171: 52–56. 17098388 10.1016/j.forsciint.2006.10.005

[28] Grobosch T, Angelow B, Schönberg L, et al. Acute bromadiolone intoxication. J Anal Toxicol. 2006;30:281–286. 16803669 10.1093/jat/30.4.281

[29] Morgan BW, Tomaszewski C, Rotker I Spontaneous hemoperitoneum from brodifacoum overdose. Am J Emerg Med. 1996;14:656–659. 8906764 10.1016/S0735-6757(96)90082-0

[30] Kanabar D, Volans G Accidental superwarfarin poisoning in children-less treatment is better. Lancet. 2002;360:963. 12383662 10.1016/S0140-6736(02)11120-2

